# A Symptom-Based Algorithm for Rapid Clinical Diagnosis of COVID-19 in Adults With High-Risk Exposure

**DOI:** 10.7759/cureus.89589

**Published:** 2025-08-07

**Authors:** Shinji Inaba, Shuntaro Ikeda, Naoki Tsuda, Kyosei Sogabe, Katsuji Inoue, Naoyuki Nogami, Eiichi Ishii, Osamu Yamaguchi

**Affiliations:** 1 Department of Cardiology, Pulmonology, Hypertension, and Nephrology, Ehime University Graduate School of Medicine, Toon, JPN; 2 Department of Community Medicine, Pulmonology, and Cardiology, Ehime University Graduate School of Medicine, Toon, JPN; 3 Department of Cardiology, Imabari City Medical Association General Hospital, Imabari, JPN; 4 Department of Surgery, Imabari City Medical Association General Hospital, Imabari, JPN; 5 Department of Pediatrics, Imabari City Medical Association General Hospital, Imabari, JPN

**Keywords:** clinical diagnosis, close contact, covid-19, diagnostic testing, symptoms

## Abstract

Objectives

In Japan, clinical diagnosis based solely on symptoms, without the use of test kits, has been adopted to enable the rapid identification of individuals infected with coronavirus disease 2019 (COVID-19). A history of close contact with COVID-19 patients is a prerequisite for such symptom-based diagnosis. However, the current diagnostic criteria lack objectivity. This study aimed to develop a symptom-based algorithm stratified by vaccination status to support more reliable clinical diagnosis of COVID-19 among individuals with high-risk exposure.

Methods

This retrospective, single-center study was conducted in Japan between April 2021 and May 2022. An algorithm for predicting COVID-19 infection was developed by comparing symptoms in COVID-19-positive and COVID-19-negative individuals with high-risk exposure. Analyses were stratified by vaccination status, given its potential influence on symptom presentation.

Patients

A total of 179 individuals with high-risk exposure to COVID-19 patients were included in the analysis.

Results

The most common setting of close contact was within households or among roommates (55.3%, 99/179), followed by workplace or school settings (26.3%, 47/179). The combination of all three symptoms-fever, sore throat, and cough-demonstrated 100% specificity but low sensitivity, irrespective of vaccination status. Among vaccinated individuals, the combination of sore throat and cough was a more reliable diagnostic indicator, whereas fever was more predictive among unvaccinated individuals.

Conclusion

The symptom-based diagnostic algorithm developed in this study demonstrated a sensitivity of 65.3% and a specificity of 88.5%, approaching the diagnostic performance of rapid antigen testing. This algorithm may facilitate simple, rapid, and accessible clinical diagnosis of COVID-19 in resource-limited or high-demand settings.

## Introduction

Since the first confirmed case of coronavirus disease 2019 (COVID-19) was reported in Japan on January 15, 2020, the infection has continued to spread rapidly with increasing infectivity [1-3]. The global spread of COVID-19 has placed an unprecedented burden on healthcare systems, not only in Japan but worldwide. Rapid and accurate diagnosis is critical to preventing further transmission and minimizing the strain on healthcare infrastructure [4].

Laboratory-based diagnostic tools, including reverse transcription polymerase chain reaction (RT-PCR) and rapid antigen tests, are widely used for detecting COVID-19 [5,6]. However, their availability may be limited during periods of surging case numbers or in resource-constrained settings. Furthermore, large volumes of outpatient visits during outbreaks place a heavy burden on the healthcare system. In response to these challenges, symptom-based clinical diagnosis without confirmatory testing has been utilized in Japan, especially during outbreaks [7]. This approach, which assumes a high pre-test probability due to close contact with confirmed COVID-19 patients, allows for the rapid identification of infected individuals. However, the criteria for symptom-based diagnosis are often vague, lack standardization, and may lead to diagnostic uncertainty.

Therefore, this study aimed to develop a practical and objective symptom-based algorithm to support more accurate clinical decision-making for individuals with high-risk exposure to COVID-19. Furthermore, since COVID-19 symptoms have been reported to vary depending on vaccination history [2], this study will validate symptom-based diagnostic criteria stratified by vaccination status.

## Materials and methods

Study design and setting

**Figure 1:**
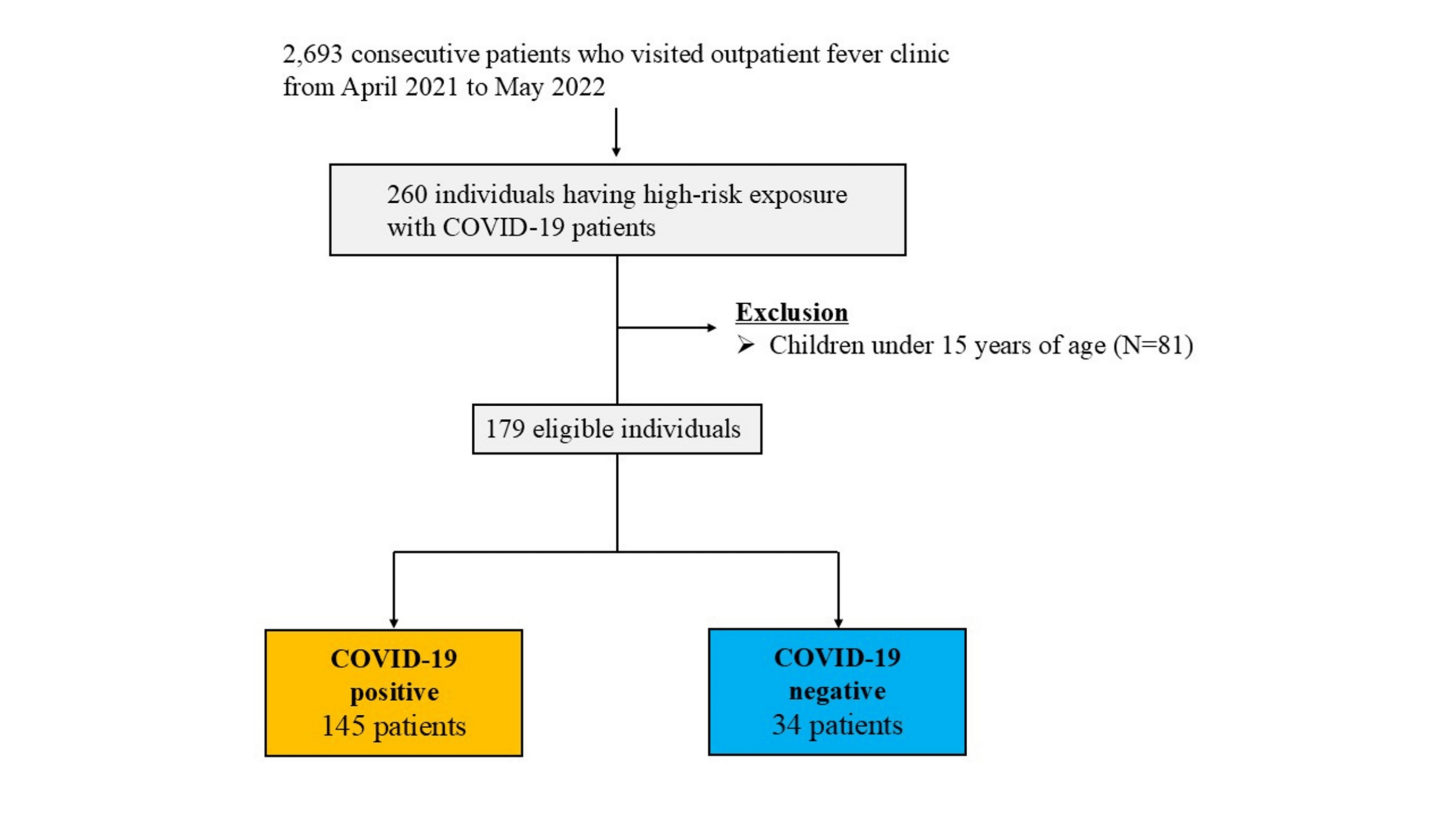
Study population flow diagram Flowchart showing participant inclusion and exclusion criteria. Of the 179 eligible individuals, 145 were diagnosed with COVID-19 infection based on RT-PCR and/or rapid antigen test results. COVID-19, coronavirus disease 2019; RT-PCR, reverse transcription polymerase chain reaction

This retrospective, single-center study was conducted at the outpatient fever clinic of Imabari City Medical Association General Hospital in Japan between April 2021 and May 2022. A total of 2,693 consecutive patients visited the clinic during the study period. Among the 2,693 patients, 114 with incomplete or missing medical records were excluded. Consequently, 2,579 patients were enrolled in the present study. Of these, 260 patients were identified as having high-risk exposure to confirmed COVID-19 cases. After excluding 81 children under 15 years of age, a final analysis was conducted on 179 patients (Figure 1).

The study protocol was approved by the Research Ethics Committee of the Ehime University Graduate School of Medicine (approval number: 2201006).

COVID-19 diagnosis was confirmed using RT-PCR tests and/or rapid antigen tests performed on nasopharyngeal swab specimens. Prior to testing, all patients were asked about their history of close contact with COVID-19 patients, vaccination status, and symptoms using a standardized questionnaire. High-risk exposure was defined as face-to-face contact within 1 m for at least 15 minutes with a person confirmed to be infected with COVID-19, regardless of mask usage [1,2]. This definition was adopted because the effectiveness of masks in preventing COVID-19 transmission remains a subject of debate. Vaccination history was defined as having received at least one dose of any COVID-19 vaccine. Fever was defined as a body temperature of ≥37.5°C at the time of clinical presentation [1,2].

Statistical analysis 

Categorical variables were presented as frequencies and percentages, while continuous variables were expressed as medians with interquartile ranges (IQRs). Fisher’s exact test was used to compare categorical variables, and the Mann-Whitney U test was applied for comparisons of continuous variables. A p-value of <0.05 was considered statistically significant. All statistical analyses were performed using EZR (Saitama Medical Center, Jichi Medical University, Saitama, Japan) [8].

## Results

Of the 179 eligible adults, 145 (81%) tested positive for COVID-19. The most common opportunities for close contact were households or among roommates (55.3%, 99/179), followed by workplace or school settings (26.3%, 47/179), and meals or conversations with COVID-19-infected patients (14.0%, 25/179). A comparison of symptom frequencies between COVID-19-positive and COVID-19-negative individuals is presented in Table 1.

**Table 1:** Comparison of symptoms between COVID-19-positive and COVID-19-negative patients Values are median (interquartile range) or number of patients (%). # indicates the statistical analysis using the Mann-Whitney U test for comparisons of continuous variables (age and temperature at clinic). Fisher’s exact test was used to compare categorical variables other than continuous variables. COVID-19, coronavirus disease 2019

	COVID-19 positive (145)	COVID-19 negative (34)	p-value
Age, years	38.0 (27.0, 49.0)	39.5 (27.8, 46.0)	0.89^#^
Sex, male	59 (40.7%)	10 (29.4%)	0.25
History of COVID-19 vaccination	108/131 (82.4%)	20/26 (76.9%)	0.58
Temperature at clinic (℃)	37.6 (36.8, 38.1)	36.8 (36.6, 37.1)	<0.001^#^
Fever (≥37.5℃ at clinic)	73/138 (52.9%)	4/33 (12.1%)	<0.001
Sore throat	107 (73.8%)	17 (50.0%)	0.012
Cough	104 (71.7%)	12 (35.3%)	<0.001
Headache	57 (39.3%)	13 (38.2%)	1
Runny nose/nasal congestion	40 (27.6%)	13 (38.2%)	0.30
Fatigue	36 (24.8%)	9 (26.5%)	0.83
Sputum	34 (23.4%)	4 (11.8%)	0.17
Arthralgia	14 (9.7%)	0 (0%)	0.08
Loss of appetite	14 (9.7%)	2 (5.9%)	0.74
Chills	8 (5.5%)	0 (0%)	0.36
Diarrhea	9 (6.2%)	0 (0%)	0.21
Nausea/vomiting	7 (4.8%)	1 (2.9%)	1
Shortness of breath	6 (4.1%)	3 (8.8%)	0.38
No symptoms	5 (3.4%)	2 (5.9%)	0.62
Abdominal pain	1 (0.7%)	0 (0%)	1
Myalgia	4 (0.7%)	0 (0%)	1
Gastralgia	3 (2.1%)	0 (0%)	1
Loss of taste	1 (0.7%)	0 (0%)	1
Loss of smell	1 (0.7%)	0 (0%)	1
Cystitis symptom	0 (0%)	0 (0%)	NA
Constipation	0 (0%)	0 (0%)	NA

Fever, sore throat, and cough were significantly more common among COVID-19-positive individuals. Among the 157 individuals with confirmed vaccination status (excluding two with unknown vaccination history), 128 (81.5%) had received at least one dose of a COVID-19 vaccine. Differences in symptom presentation between vaccinated and unvaccinated individuals are summarized in Table 2. Among vaccinated individuals, fever, sore throat, and cough were significantly more frequent in COVID-19-positive individuals compared to those who were COVID-19-negative. In contrast, among unvaccinated individuals, only fever was significantly more common in those who tested positive (Table 2).

**Table 2:** Comparison of symptoms between COVID-19-positive and COVID-19-negative patients among vaccinated and non-vaccinated groups Values are median (interquartile range) or number of patients (%). # indicates the statistical analysis using the Mann-Whitney U test for comparisons of continuous variables (age and temperature at clinic). Fisher’s exact test was used to compare categorical variables other than continuous variables. COVID-19, coronavirus disease 2019

	Vaccination (+)		p-value	Vaccination (-)		p-value
	COVID-19-positive	COVID-19-negative		COVID-19-positive	COVID-19-negative	
	(108)	(20)		(23)	(6)	
Age, years	40.0 (31.0, 51.0)	37.5 (24.3, 51.3)	0.48^#^	29.0 (21.5, 43.0)	34.0 (28.3, 39.0)	0.35^#^
Sex, male	43 (39.8%)	7 (35.0%)	0.81	13 (56.5%)	2 (33.3%)	0.39
Temperature at clinic (℃)	37.6 (36.8, 38.1)	36.8 (36.5, 37.1)	0.004^#^	38.2 (37.5, 38.4)	36.7 (36.6, 37.2)	0.008^#^
Fever (≥37.5℃ at clinic)	47/103 (45.6%)	2 (10.0%)	0.003	17/21 (81.0%)	1 (16.7%)	0.008
Sore throat	84 (77.8%)	10 (50.0%)	0.014	15 (65.2%)	3 (50.0%)	0.65
Cough	81 (75.0%)	6 (30.0%)	<0.001	13 (56.5%)	2 (33.3%)	0.39
Headache	39 (36.1%)	8 (40.0%)	0.80	11 (47.8%)	3 (50.0%)	1
Runny nose/nasal congestion	30 (27.8%)	9 (45.0%)	0.18	7 (30.4%)	3 (50.0%)	0.63
Fatigue	25 (23.1%)	5 (25.0%)	1	5 (21.7%)	2 (33.3%)	0.61
Sputum	26 (24.1%)	2 (10.0%)	0.24	5 (21.7%)	1 (16.7%)	1
Arthralgia	11 (10.2%)	0 (0%)	0.21	3 (13.0%)	0 (0/%)	1
Loss of appetite	11 (10.2%)	1 (5.0%)	0.69	2 (8.7%)	0 (0/%)	1
Chills	5 (4.6%)	0 (0%)	1	2 (8.7%)	0 (0/%)	1
Diarrhea	7 (6.5%)	0 (0%)	0.60	1 (4.3%)	0 (0/%)	1
Nausea/vomiting	6 (5.6%)	0 (0%)	0.59	1 (4.3%)	1 (16.7%)	0.38
Shortness of breath	4 (3.7%)	2 (10.0%)	0.24	1 (4.3%)	1 (16.7%)	0.38
No symptoms	4 (3.7%)	1 (5.0%)	0.58	0 (0/%)	1 (16.7%)	0.21
Abdominal pain	1 (0.9%)	0 (0%)	1	0 (0/%)	0 (0/%)	NA
Myalgia	3 (2.8%)	0 (0%)	1	1 (4.3%)	0 (0/%)	1
Gastralgia	2 (1.9%)	0 (0%)	1	1 (4.3%)	0 (0/%)	1
Loss of taste	1 (0.9%)	0 (0%)	1	0 (0/%)	0 (0/%)	NA
Loss of smell	1 (0.9%)	0 (0%)	1	0 (0/%)	0 (0/%)	NA
Cystitis symptom	0 (0%)	0 (0%)	NA	0 (0/%)	0 (0/%)	NA
Constipation	0 (0%)	0 (0%)	NA	0 (0/%)	0 (0/%)	NA

**Figure 2:**
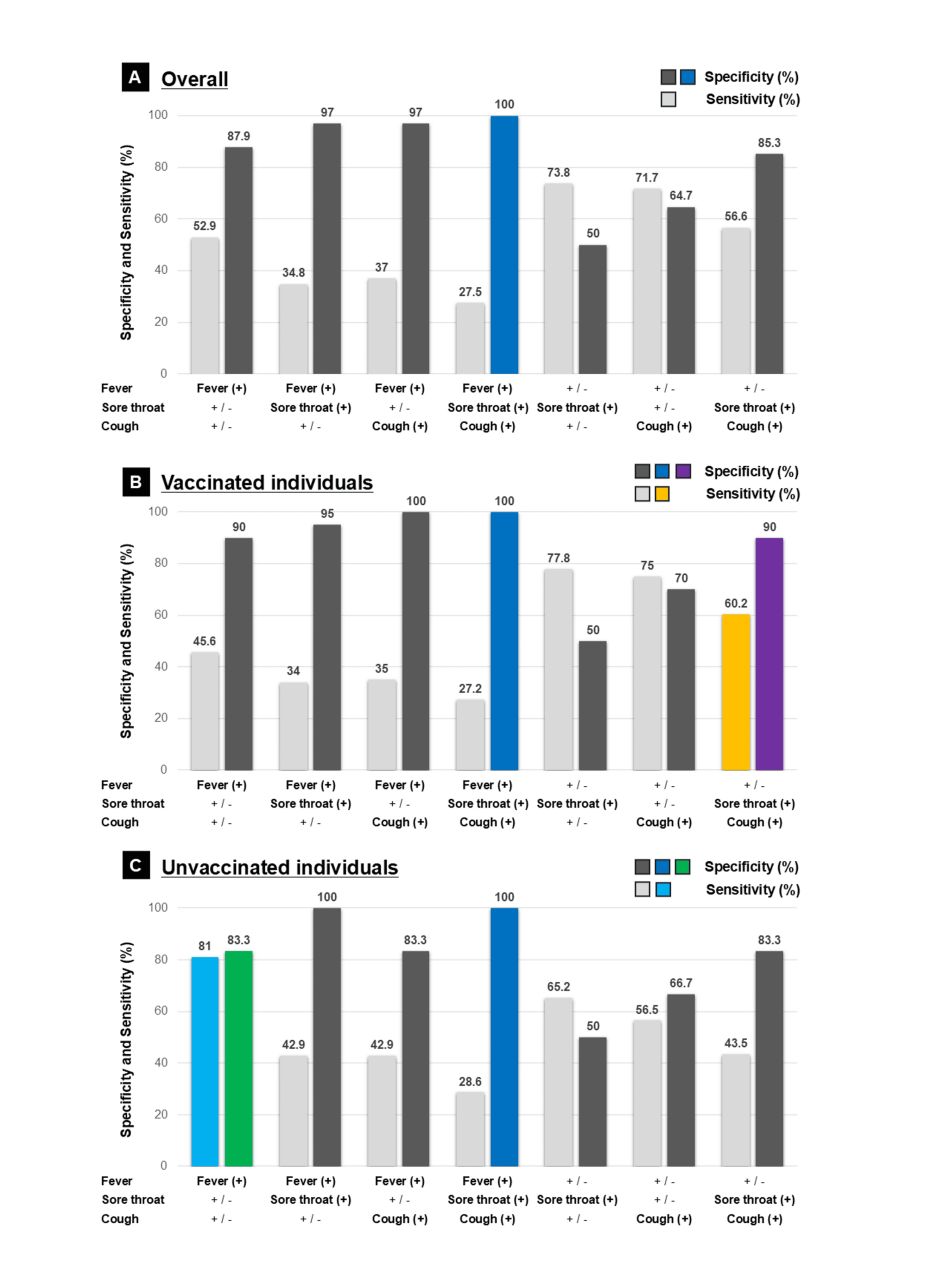
Sensitivity and specificity of symptom combinations for diagnosing COVID-19 (A) Overall population. (B) Vaccinated individuals. (C) Unvaccinated individuals. Sensitivity and specificity were calculated for different combinations of fever, sore throat, and cough. The combination of all three symptoms demonstrated the highest specificity (100%) (blue bars) but low sensitivity in all groups. Among vaccinated individuals, the combination of sore throat and cough yielded both high sensitivity (yellow bar) and specificity (purple bar). In unvaccinated individuals, fever was the most predictive symptom (sensitivity: light blue bar, specificity: green bar). *The symbol "+/-" indicates that it does not matter whether symptoms can be present or absent.

The diagnostic performance of symptom combinations - fever, sore throat, and cough - is shown in Figure 2.

**Figure 3:**
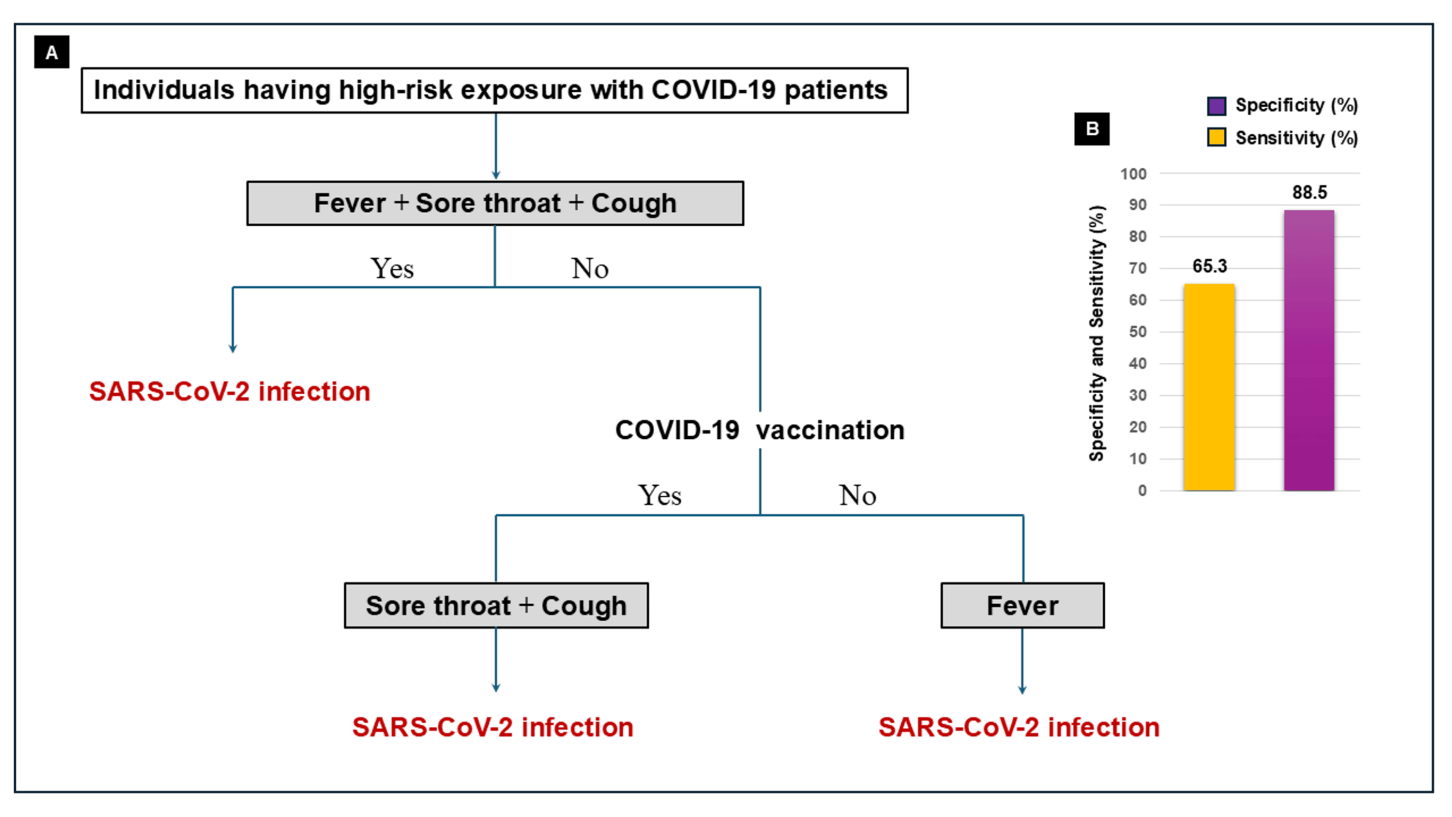
Symptom-based diagnostic algorithm for COVID-19 A simple clinical diagnostic algorithm for identifying COVID-19 in individuals with high-risk exposure. The model incorporates vaccination status and evaluates the presence of fever, sore throat, and cough. This algorithm achieved a sensitivity of 65.3% and a specificity of 88.5%, comparable to rapid antigen testing.

The combination of fever with sore throat and cough demonstrated high specificity regardless of vaccination status, reaching 100% specificity when all three symptoms were present. However, sensitivity remained low (<50%) for all combinations involving fever. In vaccinated individuals, the combination of sore throat and cough demonstrated both high sensitivity and specificity (Figure 2B), whereas among unvaccinated individuals, fever was the most predictive symptom (Figure 2C). A symptom-based diagnostic algorithm is presented in Figure 3.

This algorithm achieved a specificity of 88.5% and a sensitivity of 65.3%, approximating the diagnostic accuracy of rapid antigen testing.

## Discussion

In this study, we evaluated the diagnostic utility of simple symptom combinations for identifying COVID-19 among individuals with high-risk exposure. The symptom-based algorithm we developed for rapid clinical diagnosis demonstrated that the combination of fever, sore throat, and cough yielded a high diagnostic specificity of 88.5%. This algorithm, which does not require the use of diagnostic test kits, offers several practical advantages as follows: (1) rapid diagnosis during large-scale outbreaks can alleviate the burden on healthcare and public health systems by reducing congestion in testing facilities and clinics, (2) early identification of infected individuals enables prompt self-isolation and treatment, thereby helping to prevent further transmission, and (3) symptom-based diagnosis may reduce healthcare costs by minimizing dependence on testing resources, which can be limited in high-demand or resource-constrained settings.

Diagnostic accuracy of RT-PCR and antigen tests for COVID-19

Among the available diagnostic tools for severe acute respiratory syndrome coronavirus 2 (SARS-CoV-2), RT-PCR has been widely recognized as the gold standard due to its high analytical sensitivity and specificity regardless of symptomatic and asymptomatic individuals. Although the diagnostic accuracy of RT-PCR varies across studies, a systematic review reported an average sensitivity of approximately 70% and specificity of 95% for RT-PCR assays [5,9]. The variability in sensitivity is largely attributable to the timing of specimen collection since SARS-CoV-2 exposure, with earlier or later sampling potentially leading to false-negative results [10]. Therefore, even RT-PCR, despite its gold standard status, requires careful interpretation in the context of clinical and epidemiological information.

The diagnostic accuracy of antigen test kits is lower than that of RT-PCR, particularly in asymptomatic individuals with low viral loads [6]. According to a Cochrane review, the average sensitivity of antigen tests is 56.2%, while specificity remains consistently high across populations [6]. However, antigen test kits are valuable as screening tools due to their simplicity and rapid determination compared to RT-PCR testing. Additionally, antigen testing does not require specialized laboratory equipment and is less expensive per test. The clinical diagnostic algorithm proposed in this study shares several advantages with antigen testing, including cost-effectiveness and rapid results. However, similar to antigen testing, its lower sensitivity may lead to false-negative results. Therefore, patients at high risk for severe diseases, such as older adults or those presenting with prominent symptoms, should undergo confirmatory RT-PCR testing even if they initially test negative using the symptom-based algorithm.

Differences in clinical symptoms between vaccinated and unvaccinated individuals with COVID-19

Previous studies have demonstrated that COVID-19 vaccination not only reduces the risk of infection but also significantly attenuates disease severity [11]. However, emerging evidence suggests that vaccination status may influence the clinical presentation of COVID-19, potentially affecting the diagnostic utility of individual symptoms [2]. Therefore, symptom-based diagnostic strategies should take vaccination history into account to improve diagnostic accuracy. Fever is the most frequently reported symptom of COVID-19 [1,2,12]. However, our previous findings indicated that COVID-19 vaccination reduces both the frequency and severity of fever in adults with SARS-CoV-2 infection. In contrast, the prevalence of sore throat and cough was higher in vaccinated individuals compared to unvaccinated individuals [2]. The mechanisms underlying these symptom differences remain unclear. However, hypotheses such as vaccine-associated enhanced disease (VAED) or antibody-dependent enhancement (ADE) have been proposed [13,14]. Furthermore, repeated administration of mRNA vaccines has been suggested to transiently suppress immune function [15], which may influence the clinical manifestations observed in vaccinated individuals.

High infectivity of COVID-19

This clinical diagnostic algorithm is specifically applicable to individuals with documented close contact with COVID-19 cases. SARS-CoV-2 is characterized by a high transmission rate, particularly when compared with other respiratory viruses. Notably, the Omicron variant has demonstrated significantly enhanced transmissibility [3]. Close contact with individuals infected with SARS-CoV-2 markedly increases the risk of transmission. Therefore, early identification of infected individuals and prompt social isolation are essential for controlling the spread of the virus. In our previous research, high-risk exposure to confirmed COVID-19 patients was identified as an independent predictor of infection, with an odds ratio of 23.1. In the present cohort, 47.1% of COVID-19-positive individuals had a history of close contact with COVID-19-infected patients, consistent with previous findings [1,16-18]. Household settings represent one of the highest-risk environments for SARS-CoV-2 transmission due to prolonged and repeated exposure. Furthermore, several studies have reported that close contact with symptomatic individuals results in higher secondary transmission rates than contact with asymptomatic individuals [18]. Behavioral modifications to reduce close contact, especially in high-risk environments, are recommended when infection is suspected by clinical diagnostic algorithms. Thus, the findings of this study may contribute to efforts to prevent further spread of COVID-19.

Differentiation from other infectious diseases such as influenza

Since outbreaks of both COVID-19 and influenza may occur simultaneously, distinguishing between the two is clinically important. As both diseases are highly contagious, a history of close contact with infected individuals plays a critical role in diagnosis. In Japan, clinical diagnoses of not only COVID-19 but also influenza are often made based on symptoms alone, without the use of diagnostic test kits. Influenza is known to have a more rapid onset of symptoms than COVID-19, typically presenting with high fever, joint pain, and general fatigue, making clinical differentiation possible in some cases [19]. However, the diagnostic algorithm used in this study was not developed to differentiate between influenza and COVID-19. Furthermore, this cohort cannot adequately address this issue, as the study period coincided with a time of minimal influenza activity. Therefore, further research is required to clarify this issue.

The epidemic periods of both viruses often overlap, and co-infection within the same individual is also possible. Additionally, distinguishing COVID-19 pneumonia from community-acquired pneumonia (CAP) remains challenging, as the clinical presentations of CAP are variable and may overlap with those of COVID-19. Given these diagnostic complexities, RT-PCR testing might be recommended over antigen testing or clinical diagnosis alone, particularly when there is a history of close contact with a confirmed COVID-19 case.

Limitations

This study has several limitations. First, the retrospective single-center design limits the generalizability of the findings and introduces the potential for selection bias. Therefore, prospective validation in diverse populations and healthcare settings is necessary. Second, symptom profiles may vary with the emergence of new SARS-CoV-2 variants [20]. Although the majority of infections during the study period were attributed to the Omicron variant, revalidation of the algorithm may be necessary as novel variants become dominant. Third, the diagnostic reference standard included antigen tests, which are known to have lower sensitivity compared to RT-PCR. This may have resulted in underdiagnosis of COVID-19 cases and affected the accuracy of the algorithm. Despite these limitations, the study provides meaningful insights into the feasibility and utility of symptom-based clinical diagnosis for COVID-19, particularly in individuals with high-risk exposure or in settings with limited testing capacity.

## Conclusions

In this study, we developed a simple, symptom-based diagnostic algorithm for identifying COVID-19 infection among individuals with high-risk exposure. Importantly, the algorithm showed differential predictive value depending on vaccination status: sore throat and cough were more indicative in vaccinated individuals, while fever was more predictive among the unvaccinated. This diagnostic approach, which facilitates rapid clinical decision-making without the need for testing kits, may help reduce the burden on healthcare systems and limit viral transmission through early identification and isolation. Our findings highlight the practical utility of symptom-based diagnosis in settings with limited testing resources or during large-scale outbreaks, particularly for individuals with close contact exposure to confirmed COVID-19 patients.

## References

[1] Inaba S, Nakao Y, Ikeda S (2023). Simple symptom-based prediction of COVID-19: a single-center study of outpatient fever clinic in Japan. Cureus.

[2] Inaba S, Ikeda S, Fujiwara Y (2024). Diagnosis of COVID-19: is fever the best indicator of COVID-19 in vaccinated SARS-CoV-2-positive adults?. Cureus.

[3] Chen J, Wang R, Gilby NB, Wei GW (2022). Omicron variant (B.1.1.529): infectivity, vaccine breakthrough, and antibody resistance. J Chem Inf Model.

[4] Vandenberg O, Martiny D, Rochas O, van Belkum A, Kozlakidis Z (2021). Considerations for diagnostic COVID-19 tests. Nat Rev Microbiol.

[5] Arevalo-Rodriguez I, Buitrago-Garcia D, Simancas-Racines D (2020). False-negative results of initial RT-PCR assays for COVID-19: a systematic review. PLoS One.

[6] Dinnes J, Deeks JJ, Berhane S (2021). Rapid, point-of-care antigen and molecular-based tests for diagnosis of SARS-CoV-2 infection. Cochrane Database Syst Rev.

[7] (2025). Ministry of Health, Labour and Welfare, Japan. Handling of COVID-19 “presumed positive” cases based on clinical judgment without testing. https://www.mhlw.go.jp/stf/covid-19/kenkou-iryousoudan.html.

[8] Kanda Y (2013). Investigation of the freely available easy-to-use software 'EZR' for medical statistics. Bone Marrow Transplant.

[9] Watson J, Whiting PF, Brush JE (2020). Interpreting a covid-19 test result. BMJ.

[10] Kucirka LM, Lauer SA, Laeyendecker O, Boon D, Lessler J (2020). Variation in false-negative rate of reverse transcriptase polymerase chain reaction-based SARS-CoV-2 tests by time since exposure. Ann Intern Med.

[11] Thompson MG, Burgess JL, Naleway AL (2024). Prevention and attenuation of Covid-19 with the BNT162b2 and mRNA-1273 vaccines. N Engl J Med.

[12] Guan WJ, Ni ZY, Hu Y (2020). Clinical characteristics of coronavirus disease 2019 in China. N Engl J Med.

[13] Ikewaki N, Kurosawa G, Levy GA, Preethy S, Abraham SJ (2023). Antibody dependent disease enhancement (ADE) after COVID-19 vaccination and beta glucans as a safer strategy in management. Vaccine.

[14] Halstead SB, Katzelnick L (2020). COVID-19 vaccines: should we fear ADE?. J Infect Dis.

[15] Uversky VN, Redwan EM, Makis W, Rubio-Casillas A (2023). IgG4 antibodies induced by repeated vaccination may generate immune tolerance to the SARS-CoV-2 spike protein. Vaccines (Basel).

[16] Bi Q, Wu Y, Mei S (2020). Epidemiology and transmission of COVID-19 in 391 cases and 1286 of their close contacts in Shenzhen, China: a retrospective cohort study. Lancet Infect Dis.

[17] Trübner F, Steigert L, Echterdiek F (2021). Predictors of COVID-19 in an outpatient fever clinic. PLoS One.

[18] Madewell ZJ, Yang Y, Longini IM Jr, Halloran ME, Dean NE (2022). Household secondary attack rates of SARS-CoV-2 by variant and vaccination status: an updated systematic review and meta-analysis. JAMA Netw Open.

[19] Fukui S, Inui A, Saita M, Kobayashi D, Naito T (2022). Comparison of the clinical parameters of patients with COVID-19 and influenza using blood test data: a retrospective cross-sectional survey. J Int Med Res.

[20] Laracy JC, Robilotti EV, Yan J, Lucca A, Aslam A, Babady NE, Kamboj M (2023). Comparison of coronavirus disease 2019 (COVID-19) symptoms at diagnosis among healthcare personnel before and after the emergence of the omicron variant. Infect Control Hosp Epidemiol.

